# Comparison of FibroScan-Aspartate Aminotransferase (FAST) Score and Other Non-invasive Surrogates in Predicting High-Risk Non-alcoholic Steatohepatitis Criteria

**DOI:** 10.3389/fmed.2022.869190

**Published:** 2022-04-14

**Authors:** Jae Seung Lee, Hye Won Lee, Beom Kyung Kim, Jun Yong Park, Do Young Kim, Sang Hoon Ahn, Jae Young Jang, Soo Young Park, Hyun Woong Lee, Chun Kyon Lee, Seung Up Kim

**Affiliations:** ^1^Department of Internal Medicine, Yonsei University College of Medicine, Seoul, South Korea; ^2^Institute of Gastroenterology, Yonsei University College of Medicine, Seoul, South Korea; ^3^Yonsei Liver Center, Severance Hospital, Seoul, South Korea; ^4^Department of Internal Medicine, Soonchunhyang University School of Medicine, Seoul, South Korea; ^5^Department of Internal Medicine, Kyungpook National University Hospital, Daegu, South Korea; ^6^Department of Internal Medicine, Gangnam Severance Hospital, Seoul, South Korea; ^7^Department of Internal Medicine, National Health Insurance Cooperation, Ilsan Hospital, Goyang, South Korea

**Keywords:** fatty liver, non-alcoholic fatty liver disease, non-alcoholic steatohepatitis, liver function tests, fibrosis, elasticity imaging techniques

## Abstract

Identification of non-alcoholic steatohepatitis (NASH) with high activity and fibrosis is a major priority in patients with non-alcoholic fatty liver disease. We validated the predictive value of the FibroScan-aspartate aminotransferase (FAST) score and other non-invasive fibrosis surrogates in predicting high-risk NASH criteria. This multicenter retrospective study recruited 251 biopsy-proven non-alcoholic fatty liver disease (NAFLD) patients (132 [52.6%] men) between 2011 and 2014. The FAST score was calculated using transient elastography data and aspartate aminotransferase (AST) levels. The NAFLD fibrosis score (NFS), fibrosis-4 index (FIB-4), and AST to platelet ratio index (APRI) were calculated using biochemical data. The area under the receiver operating characteristic curves (AUCs) of the FAST score, liver stiffness, NFS, FIB-4, and APRI were 0.752, 0.718, 0.609, 0.650, and 0.722 for NAFLD activity score (NAS) ≥5 (*n* = 117, 46.6%); 0.788, 0.754, 0.649, 0.701, and 0.747 for fatty liver inhibition of progression-NASH with histologic activity ≥3 (*n* = 202, 80.5%); 0.807, 0.806, 0.691, 0.732, and 0.760 for severe disease with activity ≥3 and/or fibrosis ≥3 (*n* = 132, 52.6%); and 0.714, 0.812, 0.748, 0.738, and 0.669 for NASH with NAS ≥4 and fibrosis ≥2 (*n* = 70, 27.9%), respectively. The FAST score had the highest AUC for the most high-risk NASH criteria, except for in predicting NAS ≥4 and fibrosis ≥2. The liver stiffness value showed consistently acceptable performance in predicting all high-risk NASH criteria. The FAST score has acceptable performance in identifying high-risk NASH. However, liver stiffness alone was not inferior to the FAST score.

## Introduction

The global prevalence of non-alcoholic fatty liver disease (NAFLD) is estimated to be 24%, which is now an emerging cause of advanced liver diseases such as cirrhosis and hepatocellular carcinoma (HCC) (1–4). The prevalence of NAFLD in the Republic of Korea also increased rapidly from 18.6% in 1998–2001 to 21.5% in 2016–2017, with an increasing prevalence of obesity and diabetes (5). Approximately 25–40% of patients with NAFLD progress to non-alcoholic steatohepatitis (NASH), which exhibits more aggressive histologic findings with necroinflammatory activity and has an increased risk of liver fibrosis (6). The priority of identifying patients with NASH and significant fibrosis arises from their prognosis, with an increased risk of cirrhosis and HCC (2, 7). Moreover, the lack of effective and evidence-based pharmacotherapy necessitated various clinical trials for NAFLD that require the identification of patients with advanced inflammation and fibrosis. Although liver biopsy is the gold standard for evaluation, histological assessment is impractical because of its invasiveness, cost, sampling error, and interobserver variability. Therefore, efforts have been made to estimate histologic inflammation and fibrosis through non-invasive methods using biomarkers or imaging techniques (8–11).

Recently, Newsome et al. suggested the FibroScan-aspartate aminotransferase (FAST) score for the non-invasive identification of patients with NASH with NAFLD activity score (NAS) ≥4 and fibrosis stage ≥2, which represent high-risk NASH (12). The score, combining liver stiffness (LS) and controlled attenuation parameter (CAP) values using transient elastography (TE) with AST levels, showed high performance (area under the receiver operating characteristic curve [AUC]: 0.74–0.95) in the derivation and validation cohorts. Moreover, recent studies have supported that the FAST score is reliable in stratifying high-risk NASH in Japanese (AUC: 0.76) and US veterans (AUC: 0.75), regardless of the TE probe type (13, 14). However, the method of determining high-risk NASH can be different, and studies directly comparing the FAST score with other non-invasive surrogates such as LS value by TE, NAFLD fibrosis score (NFS), fibrosis index based on four factors (FIB-4), and AST to platelet ratio index (APRI) in predicting high-risk NASH according to various criteria are lacking (15).

Therefore, this study aimed to validate the performance of the FAST score in predicting high-risk NASH defined by various criteria and compare its performance to that of other non-invasive surrogates, using the biopsy-proven NAFLD cohort.

## Materials and Methods

### Patients' Eligibility

Patients with biopsy-proven NAFLD were recruited between January 2011 and December 2020 through a retrospective review using data from five tertiary medical centers in the Republic of Korea (Yonsei University Severance Hospital, Yonsei University Gangnam Severance Hospital, Soonchunhyang University Seoul Hospital, Kyungpook National University Hospital, and National Health Insurance Cooperation Ilsan Hospital). The inclusion criteria were as follows: (1) age ≥19 years, (2) histological findings of hepatic steatosis, (3) no significant alcohol intake (>30 g/day for men and 20 g/day for women), and (4) TE performed with an M probe on the day of liver biopsy. The exclusion criteria were as follows: (1) other causes of chronic hepatitis, such as viral hepatitis B and C; (2) any histological findings suggesting other chronic liver disease or secondary to other etiologies (e.g., autoimmune hepatitis, congestive hepatopathy, primary biliary cholangitis, primary sclerosing cholangitis, and Wilson's disease); (3) previous hepatic or other malignancies; (4) any signs of acute hepatitis or liver failure, defined as AST level >300 IU/L, alanine aminotransferase level >300 IU/L, total bilirubin level >3.0 mg/dL, or serum albumin level <2.5 g/dL; (5) drug exposure that can induce secondary hepatic steatosis (for example, corticosteroids, tamoxifen, and amiodarone); (6) TE assessment failure or unreliable TE results; and (7) insufficient clinical data.

The study protocol was in accordance with the ethical guidelines of the 1975 Declaration of Helsinki. The need for written informed consent was waived because of the retrospective nature of the study. The study procedure was approved by the institutional review board of each institute (IRB No. 4-2021-0239).

### Liver Biopsy and Histological Assessment

All patients underwent ultrasonography-guided liver biopsy using a 19-gauge biopsy needle. To acquire adequate samples, at least two tissues, approximately 2 cm in length, were obtained from each patient. Liver biopsies were routinely formalin-fixed, paraffin-embedded, and assessed by individual pathologists at each institute.

Steatosis (0–3), ballooning (0–2), and lobular inflammation (0–3) were scored to calculate the NAS using the NASH Clinical Research Network (NASH CRN) scoring system (16). The NAS was the sum of steatosis, ballooning, and lobular inflammation grades and ranged from 0 to 8 (16). NASH was defined in two ways: one using NAS ≥5, which is known as a criterion for definite NASH (16), and the other using the fatty liver inhibition of progression (FLIP) definition as the presence of steatosis, hepatocyte ballooning, and lobular inflammation with at least 1 point for each category (FLIP-NASH) (17). Steatosis, activity, and fibrosis (SAF) scores were calculated through the separate assessment of the grade of steatosis (S0–S3), activity (A0–A4 through the addition of ballooning and lobular inflammation), and the stage of fibrosis (F0–F4 with a single modification of pooling the three substages [1a, 1b, and 1c]) according to the NASH CRN (17).

### TE Assessment

At each hospital, TE (EchoSens, Paris, France) was performed by experienced operators who had conducted at least 500 examinations. Patients were examined after overnight fasting using M probes due to the limited availability of the XL probe in only one institute. LS (kPa) and CAP (dB/m) measurements were recorded until 10 valid measurements were obtained for each patient. The median value was considered representative of the elastic modulus of the liver. Only procedures with at least 10 valid measurements, a success rate of at least 60%, and an interquartile range (IQR) to median value ratio of 30% were considered reliable (18–20).

### FAST Score and Other Non-invasive Surrogates

The patients' body mass index (BMI; kg/m2) and biochemical data were obtained at the time of liver biopsy. The FAST score was calculated according to a previously reported formula using recently measured LS, CAP, and AST levels. Using the patients' biochemical data, individual NFS, FIB-4, and APRI were also calculated according to a previously reported formula (21–23).


$$\begin{array}{l} FAST=\frac{e^{-1.65\;+\;1.07\times \ln\left(LS\left[kPa\right]\right)\;+\;2.66\times 10^{-8}\times {CAP\left(dB/m\right)}^{3}\;-\;63.3\times {AST\left(IU/L\right)}^{-1}}}{{1\;+\;e}^{-1.65\;+\;1.07\times \ln\left(LS\left[kPa\right]\right)\;+\;2.66\times 10^{-8}\times {CAP\left(dB/m\right)}^{3}\;-\;63.3\times {AST\left(IU/L\right)}^{-1}}} \end{array}$$

### Endpoints

The main outcome was the diagnosis of high-risk NASH. The outcomes were assessed in various ways, as suggested in the literature: (1) definite NASH (NAS ≥5) according to the NASH CRN (16), (2) severe FLIP-NASH (with activity ≥A3) (17, 24), (3) severe disease according to the SAF scoring system (severe SAF, activity ≥A3, and/or fibrosis ≥F3) (17, 24), and (4) FLIP-NASH with NAS ≥4 and fibrosis ≥2 (NASH + NAS ≥4 + F ≥2) according to a previous study that suggested the FAST score (12).

### Statistical Analysis

Data were expressed as the median and IQR for quantitative data and as numbers with percentages in parentheses for qualitative data, as appropriate. The significance of differences between variables was evaluated using Student's *t*-test or the Mann–Whitney U test (continuous variables) and the chi-square or Fisher's exact test (categorical variables). Ordinal logistic regression analyses were performed to evaluate the relationship between each non-invasive fibrosis surrogate and each histologic finding (e.g., steatosis, ballooning, lobular inflammation, and fibrosis). The surrogates with higher McFadden's pseudo R-squared values (*R*^2^), higher χ^2^ values through the likelihood ratio, and lower values for Akaike information criteria (AIC) were considered to have a better fit to the scores of the histologic stages. The predictive performance of the FAST score and other non-invasive surrogates for histologic findings and each outcome were assessed using the AUC. AUCs were compared using the calculated 95% confidence intervals (CIs) using the DeLong test. Optimal cutoff values were chosen to maximize the sum of the sensitivity and specificity of the Youden index. The sensitivity, specificity, positive predictive value (PPV), negative predictive value (NPV), and accuracy of each surrogate for each outcome were calculated using the cutoff value.

All statistical analyses were conducted using SPSS version 26.0 for Windows (IBM Corp., Armonk, NY, USA) and R package (version 4.1.1, http://cran.r-project.org/). Two-sided *P* < 0.05 were considered statistically significant.

## Results

### Baseline Characteristics

**Figure 1:**
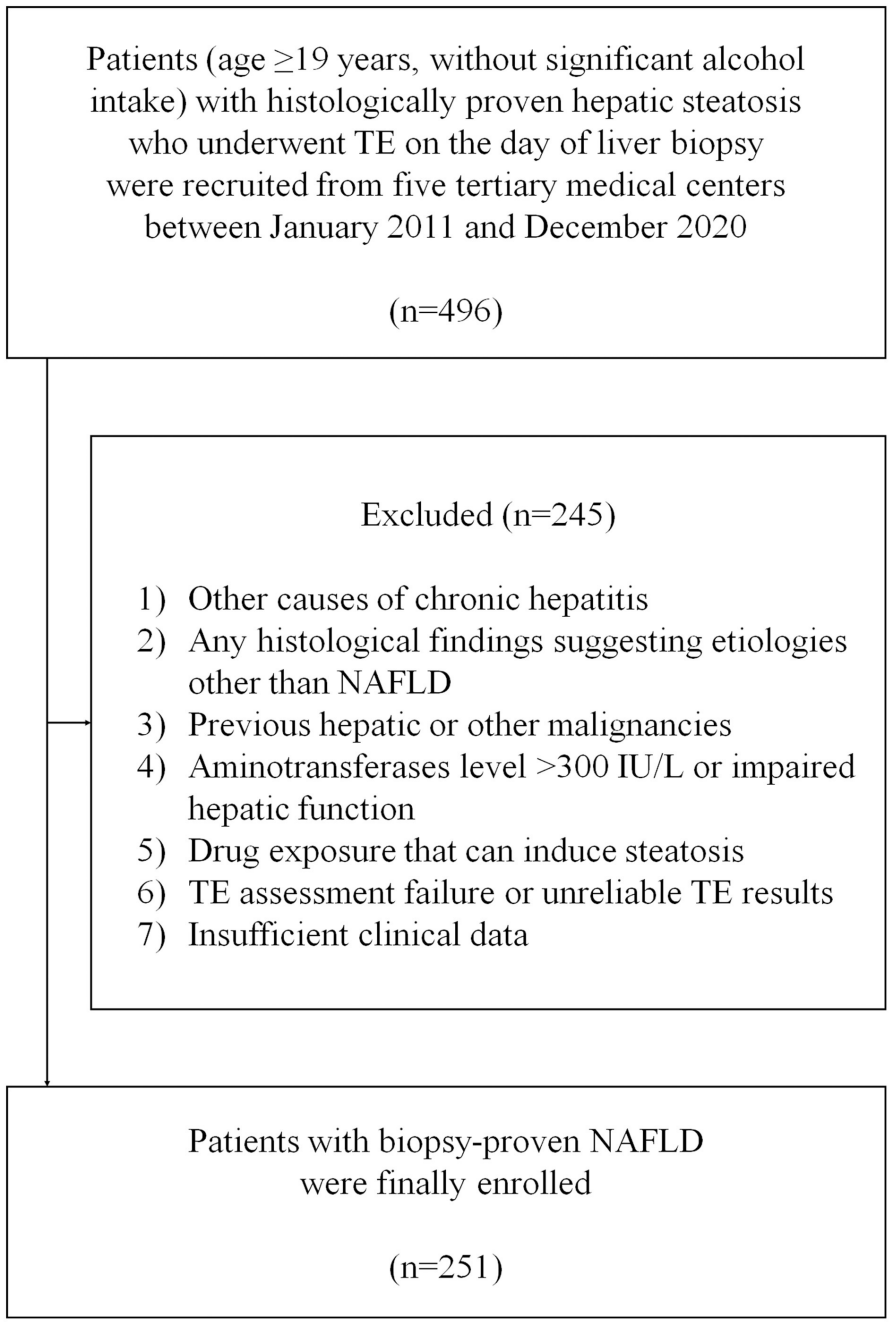
Flowchart of the study. NAFLD, non-alcoholic fatty liver disease; NASH, non-alcoholic steatohepatitis; TE, transient elastography.

A flowchart of patient selection is summarized in Figure 1. A total of 496 patients with biopsy-proven NAFLD were considered eligible. After excluding 245 patients who met the exclusion criteria, 251 patients with NAFLD were finally included.

The median age of the patients (men: 132 [52.6%]) was 44 (IQR: 34–56) years. The proportion of patients with BMI >25 kg/m2 and 30 kg/m2 was 81.7% (*n* = 205) and 37.8% (*n* = 95), respectively. Diabetes mellitus or impaired fasting glucose and hypertension were observed in 117 (46.6%) and 78 (31.1%) patients, respectively. The median LS and CAP using TE assessment were 7.8 (IQR: 6.2–11.9) kPa and 316 (IQR: 281–342) dB/m, respectively. The median NFS, FIB-4, and APRI value using biochemical data were −1.976 (IQR: −3.367 to −0.645), 1.09 (IQR: 0.67–1.99), and 0.62 (0.37–0.94), respectively. The median calculated FAST score was 0.54 (IQR: 0.33–0.69) (Table 1). All patients underwent TE on the day of liver biopsy.

**Table 1:** Patients' baseline characteristics (*n* = 251).

**Variables**	**Values**
**Demographic data**	
Age (years)	44 (34, 56)
Male sex	132 (52.6)
BMI (kg/m^2^)	28.64 (25.80, 31.45)
>25	205 (81.7)
>30	95 (37.8)
DM or IFG	117 (46.6)
Hypertension	78 (31.1)
**Biochemical data**	
Platelet count (×10^9^/L)	246 (210, 285)
Fasting glucose (mg/dL)	103 (94, 124)
Serum creatinine (mg/dL)	0.78 (0.65, 0.92)
Serum albumin (g/dL)	4.6 (4.3, 4.8)
Total bilirubin (mg/dL)	0.7 (0.5, 0.9)
AST (IU/L)	49 (32, 73)
ALT (IU/L)	58 (33, 110)
Alkaline phosphatase (IU/L)	70 (57, 88)
Gamma-GT (IU/L)	52 (32, 76)
Total cholesterol (mg/dL)	186 (161, 215)
**Non-invasive surrogates**	
Liver stiffness (kPa)	7.8 (6.2, 11.9)
CAP (dB/m)	316 (281, 342)
NFS value	−1.976 (−3.367, −0.645)
< -1.455 / ≥0.675	152 (60.6) / 20 (8.0)
FIB-4 value	1.09 (0.67, 1.99)
<1.45 / >3.25	161 (64.1)/ 22 (8.8)
APRI value	0.62 (0.37, 0.94)
<0.5 / >1.5	97 (38.6)/ 17 (6.8)
FibroScan-AST score	0.54 (0.33, 0.69)
<0.35 / ≥0.67	69 (27.5)/ 71 (28.3)

*Values are expressed as n (%) and median (interquartile range). BMI, body mass index; DM, diabetes mellitus; IFG, impaired fasting glucose; AST, aspartate aminotransferase; AST, alanine aminotransferase; CAP, controlled attenuation parameter; NFS, non-alcoholic fatty liver disease fibrosis score; FIB-4, fibrosis index based on four factors; APRI, AST to platelet ratio index*.

### Histologic Findings

The histological information is summarized in Table 2. Grade 3 steatosis, grade 3 lobular inflammation, and grade 2 ballooning were identified in 43 (17.1%), 9 (3.6%), and 88 (35.1%) patients, respectively. Fibrosis stages 0, 1, 2, 3, and 4 were identified in 33 (13.1%), 137 (54.6%), 34 (13.5%), 33 (13.1%), and 14 (5.6%) patients, respectively.

**Table 2:** Histologic findings (*n* = 251).

**Histologic findings**	***n* (%)**
Steatosis (0/1/2/3)	104 (41.4)/104 (41.4)/43 (17.1)
Lobular inflammation (0/1/2/3)	27 (10.8)/124 (49.4)/91 (36.3)/ 9 (3.6)
Ballooning (0/1/2)	52 (20.7)/111 (44.2)/88 (35.1)
Fibrosis (0/1/2/3/4)	33 (13.1)/137 (54.6)/34 (13.5)/33 (13.1)/14 (5.6)
**End-points**	***n*** **(%)**
Definite NASH (NAS ≥5)	117 (46.6)
FLIP-NASH	227 (90.4)
Severe FLIP-NASH (activity ≥3)	202 (80.5)
Severe SAF (activity and/or fibrosis ≥3)	132 (52.6)
NASH + NAS ≥4 + fibrosis ≥2	70 (27.9)

*NAS, nonalcoholic fatty liver disease activity score; FLIP, fatty liver inhibition of progression algorithm; SAF, steatosis, activity, and fibrosis score; NASH, nonalcoholic steatohepatitis*.

Regarding endpoints, definite NASH, severe FLIP-NASH, severe SAF, and NASH + NAS ≥4 + F ≥2 were reported in 117 (46.6%), 202 (80.5%), 132 (52.6%), and 70 (27.9%) patients, respectively (Table 2).

### Correlation Between Non-invasive Surrogates and Each Histologic Finding

Ordinal logistic regression analyses of the FAST score, LS, CAP, NFS, FIB-4, and APRI for histologic steatosis, activity, and fibrosis stages are summarized in Supplementary Table 1. A significant correlation was observed between the CAP value (dB/m) and FAST score and the histologic steatosis stages (all *P* < 0.001) with low *R*^2^ values (0.043 and 0.047, respectively). For histologic activity, a significant correlation was found between all noninvasive fibrosis surrogates (all *P* < 0.05): FAST score showed the highest *R*^2^ (0.120) and the lowest AIC values (673.003) compared to those of the other surrogates (all *R*^2^ <0.1 and AIC >700, respectively). The LS value (kPa) best explained the degree of histological fibrosis stage (*P* < 0.001, *R*^2^ = 0.196, and AIC = 533.289) compared with other surrogates (all *P* < 0.001, *R*^2^ <0.2, and AIC > 600).

### Diagnostic Accuracy of Non-invasive Surrogates in Predicting the Degree of Fibrosis and Steatosis

The diagnostic accuracy of each non-invasive surrogate for the prediction of each fibrosis stage (≥F2, ≥F3, and ≥F4) is presented in Table 3. LS values showed higher AUCs in predicting ≥F2 (0.822; 95% CI, 0.767–0.876), ≥F3 (0.925; 95% CI, 0.893–0.957), and ≥F4 (0.963; 95% CI, 0.935–0.991) than FAST, NFS, FIB-4, and APRI values (especially for ≥F3, all *P* < 0.001). The cutoff values of LS with maximal Youden index were 8.7 kPa for ≥F2, 9.8 kPa for ≥F3, and 15.7 for ≥F4, respectively.

**Table 3:** Comparisons between non-invasive fibrosis surrogates for predicting histologic fibrosis stage.

	**AUC (95% CI)**	***P*-value (vs. LS)**	Cut-off^*^	**Se (%)**	**Sp (%)**	**PPV (%)**	**NPV (%)**	**Accuracy (%)**
**Fibrosis ≥F2 (*n* = 81, 32.1%)**								
LS (kPa)	0.822 (0.767, 0.876)	-	8.7	77.8	72.4	57.3	87.2	74.1
FAST	0.685 (0.615, 0.755)	<0.001	0.58	65.4	67.1	48.6	80.3	66.5
NFS	0.770 (0.710, 0.831)	0.181	−1.845	77.8	67.6	53.4	86.5	70.9
FIB-4	0.743 (0.677, 0.809)	0.051	1.73	56.8	83.5	62.2	80.2	74.9
APRI	0.646 (0.573, 0.719)	<0.001	0.7	61.7	70.0	49.5	79.3	67.3
**Fibrosis ≥F3 (*n* = 47, 18.7%)**								
LS (kPa)	0.925 (0.893, 0.957)	-	9.8	95.7	79.4	51.7	98.8	82.5
FAST	0.753 (0.675, 0.831)	<0.001	0.65	72.3	76.5	41.5	92.3	75.7
NFS	0.764 (0.692, 0.837)	<0.001	−2.122	89.4	55.9	31.8	95.8	62.2
FIB-4	0.747 (0.663, 0.830)	<0.001	1.59	72.3	75.5	40.5	92.2	74.9
APRI	0.636 (0.547, 0.724)	<0.001	0.7	66.0	65.7	30.7	89.3	65.7
**Fibrosis ≥F4 (*n* = 14, 5.6%)**								
LS (kPa)	0.963 (0.935, 0.991)	-	15.7	92.9	92.0	40.6	99.5	92.0
FAST	0.828 (0.746, 0.910)	<0.001	0.66	92.9	73.0	16.9	99.4	74.1
NFS	0.829 (0.682, 0.976)	0.087	−0.582	78.6	81.4	20.0	98.5	81.3
FIB-4	0.818 (0.671, 0.965)	0.068	2.71	71.4	89.9	29.4	98.2	88.8
APRI	0.755 (0.655, 0.855)	<0.001	0.8	85.7	63.3	12.1	98.7	64.5

* *Cut-off values obtained required maximal Youden index. AUC, area under the receiver operational characteristics curve; CI, confidence interval; Se, sensitivity; Sp, specificity; PPV, positive predictive value; NPV, negative predictive value; LS, liver stiffness; FAST, FibroScan-aspartate aminotransferase (AST) score; NFS, non-alcoholic fatty liver disease fibrosis score; FIB-4, fibrosis index based on four factors; APRI, aspartate aminotransferase to platelet ratio index*.

The AUCs of CAP for ≥S2 (*n* = 147, 58.3%) and ≥S3 (*n* = 43, 17.1%) were 0.685 (95% CI, 0.618–0.752; cutoff, 280 dB/m) and 0.570 (95% CI, 0.482–0.658; cutoff, 281 dB/m), respectively.

### Diagnostic Accuracy of the FAST Score and Other Non-invasive Surrogates for High-Risk NASH

The diagnostic accuracies of all surrogates for high-risk NASH are summarized in Table 4. The FAST score showed the highest AUC in predicting definite NASH, severe FLIP-NASH, and severe SAF. For definite NASH (NAS ≥5), the AUC of the FAST score (cutoff: 0.48) was 0.752 (95% CI: 0.692–0.812), which was statistically similar to those of LS (0.718, *P* = 0.202) and APRI (0.722, *P* = 0.209) and significantly higher than those of the NFS (0.609, *P* = 0.002) and FIB-4 (0.650, *P* = 0.009). For severe FLIP-NASH, the AUC of the FAST score (cutoff: 0.58) was 0.788 (95% CI: 0.732–0.843), which was statistically similar to those of LS (0.754, *P* = 0.198) and APRI (0.747, *P* = 0.087) and significantly higher than those of the NFS (0.649, *P* = 0.002) and FIB-4 (0.701, *P* = 0.023). For severe SAF, the AUC of the FAST score (cutoff: 0.64) was 0.807 (95% CI: 0.753–0.860), which was similar to that of LS (0.806, *P* = 0.971) and significantly higher than those of the NFS (0.691, *P* = 0.009), FIB-4 (0.732, *P* = 0.048), and APRI (0.760, *P* = 0.046).

**Table 4:** Diagnostic accuracy of non-invasive surrogates in predicting high-risk NASH.

	**AUC (95% CI)**	***P*-value (vs. FAST)**	Cut-off^*^	**Se (%)**	**Sp (%)**	**PPV (%)**	**NPV (%)**	**Accuracy (%)**
**Definite NASH (NAS ≥5) (*n* = 117, 46.6%)**								
FAST	0.752 (0.692, 0.812)	-	0.48	80.3	58.2	62.7	77.2	68.5
LS (kPa)	0.718 (0.654, 0.782)	0.202	7.7	77.8	62.7	64.5	76.4	69.7
NFS	0.609 (0.539, 0.678)	0.002	−1.566	54.7	68.7	60.4	63.4	62.2
FIB-4	0.650 (0.582, 0.718)	0.009	0.90	71.8	53.7	57.5	68.6	62.2
APRI	0.722 (0.659, 0.785)	0.209	0.7	59.8	76.1	68.6	68.5	68.5
**Severe FLIP-NASH (FLIP-NASH with activity ≥3) (*n* = 202, 80.5%)**								
FAST	0.788 (0.732, 0.843)	-	0.58	65.9	78.1	74.3	70.4	72.1
LS (kPa)	0.754 (0.693, 0.814)	0.198	7.7	78.9	65.6	68.8	76.4	72.1
NFS	0.649 (0.581, 0.716)	0.002	−1.663	58.5	69.5	64.9	63.6	64.1
FIB-4	0.701 (0.636, 0.765)	0.023	1.50	51.2	81.3	72.4	63.4	66.5
APRI	0.747 (0.686, 0.808)	0.087	0.7	61.0	78.9	73.5	67.8	70.1
**Severe SAF (Activity ≥3 and/or fibrosis ≥3) (*n* = 132, 52.6%)**								
FAST	0.807 (0.753, 0.860)	-	0.64	58.3	89.9	86.5	66.0	73.3
LS (kPa)	0.806 (0.752, 0.860)	0.971	7.7	80.3	70.6	75.2	76.4	75.7
NFS	0.691 (0.626, 0.756)	0.009	−1.779	62.1	71.4	70.7	63.0	66.5
FIB-4	0.732 (0.670, 0.794)	0.048	1.50	52.3	84.9	79.3	61.6	67.7
APRI	0.760 (0.700, 0.820)	0.046	0.7	61.4	82.4	79.4	65.8	71.3
**FLIP-NASH with NAS ≥4 and fibrosis ≥2 (*n* = 70, 27.9%)**								
FAST	0.714 (0.646, 0.782)	-	0.57	69.4	67.0	45.9	84.5	67.7
LS (kPa)	0.812 (0.755, 0.869)	<0.001	7.7	90.3	57.5	46.1	93.6	66.9
NFS	0.748 (0.684, 0.813)	<0.001	−1.779	76.4	65.9	47.4	87.4	68.9
FIB-4	0.738 (0.670, 0.807)	0.021	1.59	62.5	78.2	53.6	83.8	73.7
APRI	0.669 (0.597, 0.741)	0.089	0.7	63.9	69.8	46.0	82.8	68.1

* *Cutoff values obtained required the maximal Youden index. FAST, FibroScan-aspartate aminotransferase (AST) score; LS, liver stiffness; NFS, non-alcoholic fatty liver disease (NAFLD) fibrosis score; FIB-4, fibrosis index based on four factors; APRI, AST to platelet ratio index; AUC, area under the receiver operating characteristic curve; CI, confidence interval; Se, sensitivity; Sp, specificity; PPV, positive predictive value; NPV, negative predictive value; NASH, non-alcoholic steatohepatitis; FLIP, fatty liver inhibition of progression algorithm; SAF, steatosis, activity, and fibrosis score*.

In predicting NASH + NAS ≥4 + F ≥2, the FAST score did not show a superior AUC (0.714; 95% CI, 0.646–0.782), considering the significantly higher AUCs of LS (0.812, *P* < 0.001), NFS (0.748, *P* < 0.001), and FIB-4 (0.738, *P* = 0.021). The cutoff values of the FAST score were obtained as 0.37 for 90% sensitivity and 0.79 for 90% specificity, respectively. Using the Youden index-based cutoff values of the FAST score (0.57), sensitivity, specificity, PPV, NPV, and accuracy were 69.4, 67.0, 45.9, 84.5, and 67.7%, respectively. Using the previously reported rule-out zone ( ≤ 0.35) and rule-in zone (≥0.67) of the FAST score (12), the sensitivity, specificity, PPV, NPV, and accuracy were 93.1, 35.2, 36.6, 92.6, and 51.8%, respectively, and 56.9, 77.1, 50.0, 81.7, and 71.3%, respectively.

Remarkably, LS itself showed consistently acceptable performances for all outcomes with a fixed cutoff value of 7.7 kPa using the Youden index (Table 4).

## Discussion

This multicenter retrospective cohort study validated the performance of the FAST score and other non-invasive surrogates in detecting high-risk NASH among patients with histologically confirmed NAFLD. The LS value by TE showed the best ordinal correlation (*R*^2^ = 0.196 and AIC = 533.289) and predictive performance (AUCs: 0.822–0.963) with the histologic fibrosis stages, whereas the FAST score (*R*^2^ = 0.120 and AIC = 673.033) had the best ordinal correlation with the histologic activity compared to that of the LS value only (*R*^2^ = 0.047 and AIC = 728.749). The FAST score showed acceptable predictive performance (AUCs: 0.714–0.807) for the various criteria for high-risk NASH: higher AUCs than that of the LS, NFS, FIB-4, and APRI for definite NASH, severe FLIP-NASH, and severe SAF; however, they were significantly inferior in predicting NASH + NAS ≥4 + F ≥2 compared to those of the LS, NFS, and FIB-4 (AUCs: 0.812, 0.748, and 0.738, respectively, all *P* < 0.05). Comparably, LS alone also showed consistent acceptable predictive performance (AUCs: 0.718–0.812) for all criteria and was significantly higher for NASH + NAS ≥4 + F ≥2 among the surrogates.

Our study had several clinical implications. First, our study is the first to apply the various criteria for “high-risk NASH” for the validation of non-invasive surrogates among patients with histologically confirmed NAFLD. Recent evidence suggests that the association between liver fibrosis and liver- and non-liver-related mortality in patients with NAFLD is stronger than that of NAS or its individual components (25, 26). However, considering that the high histological activity is related to the higher prevalence of metabolic risk factors and can accelerate fibrosis progression (24, 27) and the suggested data that patients with elevated NAS commonly acquire histological responses to trial medication (28), both fibrosis and activity should be included to define “high-risk NASH.” For this reason, Newsome et al. suggested the FLIP-NASH + NAS ≥4 + F ≥2 criteria that reflected the combination of high activity and significant fibrosis (12). However, it is uncertain whether these criteria affect the long-term prognosis of patients with NAFLD. Therefore, this study utilized additional known criteria for histologic NASH diagnosis, NASH with high activity, and NAFLD with high activity and/or fibrosis that were applied in the literature (16, 17, 24), and evaluated that the accuracy of each non-invasive surrogate is consistent among the various criteria.

Second, the predictive performance of various non-invasive surrogates, including the FAST score, LS, NFS, FIB-4, and APRI, was compared for the various criteria with comparable sample sizes (*n* = 251). The components of the FAST score include the LS, CAP, and AST, which may represent histologic fibrosis, steatosis, and activity, respectively (12), and its dominant performance in the separate validation cohorts compared to that of the FIB-4 and NFS was validated only for the NASH + NAS ≥4 + F ≥2 (12). Ordinal logistic regression analyses showed that the FAST score sufficiently explained the stages of histological activity. For this reason, although not significant, the FAST score showed the highest AUCs in predicting NAS ≥4, severe FLIP-NASH, and severe SAF among the surrogates. These findings suggest that the FAST score may be advantageous in predicting the histologic activity and its related NASH criteria.

However, the predictive performance of the FAST score for the NASH + NAS ≥4 + F ≥2 (AUC: 0.714) was inferior to those of LS, NFS, and FIB-4 and of previously reported AUCs [0.85 in external cohorts (12), 0.76 in the Japanese cohort (14), and 0.75 in the US veterans cohort (13)]. The low number (27.9%) of patients who met the criteria might have caused this result, although numbers were similar in the previous studies (12, 14). Moreover, the insufficient accuracy of the CAP value for histologic steatosis grade might have affected the prediction of the steatosis component of NAS ≥4. In this study, the rule-out cutoff (90% sensitivity) was 0.37, similar to 0.35 (sensitivity, 93.1%; NPV, 92.6%); however, the rule-in cutoff (90% specificity) was 0.79, far higher than 0.65 (specificity, 77.1%; PPV, 50.0%). Therefore, the FAST score may also be advantageous in accurately excluding high-risk NASH considering the similar rule-out cutoff; however, the performance to rule-in high-risk NASH and its reproducibility should be further evaluated.

Third, this study first presented the dominant predictive performance of the LS value compared to that of the FAST score. The results suggest that the LS value alone can sufficiently predict the various criteria of the “high-risk NASH” with consistently high AUCs, even for NASH + NAS ≥4 + F ≥2. In this study, the LS value showed the highest accuracy in predicting the actual histologic fibrosis stages compared to those of the NFS, FIB-4, and APRI. Moreover, the LS value showed the best accuracy for the fibrosis-added criteria (severe SAF and NASH + NAS ≥4 + F ≥2) and acceptable performance even for the activity-dominant criteria (NAS ≥5 and severe FLIP-NASH), with the consistently calculated best cutoff value (7.2 kPa) using the Youden index. Considering that this study was based on a retrospective review of real clinical data, the decision to perform further invasive procedures such as liver biopsy using only the LS value can be more convenient and reliable in clinical practice. However, we should consider that the discordance between CAP and the degree of steatosis might have caused a suboptimal AUC of FAST score for NASH + NAS ≥4 + F ≥2 in this study. Further studies are warranted to determine whether LS or FAST score is more predictable for patients with high-risk NASH.

Our study has several limitations. First, due to its retrospective nature and cross-sectional design, this study could have been subject to selection bias and may only present snapshots of NASH that could be resolved and/or progressed over time (29). Second, the availability of the XL probe in only one institute has limited further analysis regarding the probe type. In this study, 8 of 245 patients (3.3%) were excluded due to TE assessment failure. Even if the effect may be negligible considering that the previous study by Oeda et al. (14) showed a statistically similar accuracy of the FAST score between the M and XL probes in Asian patients, the exclusion of patients due to TE failure associated with high BMI or obesity may have influenced the study results. Third, the absence of a central review of histologic findings by independent pathologists blinded to the clinical data might critically affect the diagnosis of NASH by various criteria, which could disturb the predictive performance of the FAST score in this study. Fourth, further comparison using a magnetic resonance elastography (MRE)-based model, which reported higher accuracy in detecting significant fibrosis (30), was impossible due to the lack of paired MRE data.

In conclusion, the FAST score has acceptable performance in identifying high-risk NASH in Korean patients with histologically confirmed NAFLD and may help avoid unnecessary invasive procedures considering the high NPV. However, LS alone would still be effective, even slightly better than the FAST score, in identifying patients with high-risk NASH.

## Data Availability Statement

The datasets presented in this article are not publicly available due to patients' privacy. Requests to access the datasets should be directed to ksukorea@yuhs.ac.

## Ethics Statement

The studies involving human participants were reviewed and approved by Yonsei University Health System, Severance Hospital, Institutional Review Board. Written informed consent for participation was not required for this study in accordance with the national legislation and the institutional requirements.

## Author Contributions

Conceptualization, data curation, project administration, and writing—original draft by JL and SK. Formal analysis and visualization by JL. Funding acquisition and supervision by SK. Investigation, resources, and writing—review and editing by JL, HL, BK, JP, DK, SA, JJ, SP, HL, CL, and SK. All authors contributed to the article and approved the submitted version.

## Funding

This study was supported by the Basic Science Research Program through the National Research Foundation of Korea funded by the Ministry of Science, ICT and Future Planning (2019R1A2C4070136). The funders had no role in the study design, data collection and analysis, decision to publish, or preparation of the manuscript.

## Conflict of Interest

The authors declare that the research was conducted in the absence of any commercial or financial relationships that could be construed as a potential conflict of interest.

## Publisher's Note

All claims expressed in this article are solely those of the authors and do not necessarily represent those of their affiliated organizations, or those of the publisher, the editors and the reviewers. Any product that may be evaluated in this article, or claim that may be made by its manufacturer, is not guaranteed or endorsed by the publisher.
