# Supplementary material for: Comparison of FibroScan-Aspartate Aminotransferase (FAST) Score and Other Non-invasive Surrogates in Predicting High-Risk Non-alcoholic Steatohepatitis Criteria
Source: Front Med (Lausanne). 2022 Apr 14;9:869190. doi: 10.3389/fmed.2022.869190 (PMC9048204; doi:10.3389/fmed.2022.869190)
Supplement: Supplementary file 1 [file Table_1.DOCX]

Supplementary Material

# Supplementary Table

| **Supplementary Table 1**. Ordinal logistic regression of noninvasive surrogates for each histologic finding | | | | | | |
| --- | --- | --- | --- | --- | --- | --- |
|  | Coefficient (95% CI) | SE | *P* value | McFadden’s *R^2^* | Likelihood ratio (χ^2^) | AIC |
| Steatosis | | | | | | |
| FAST | 2.674 (1.584, 3.764) | 0.556 | <0.001 | 0.047 | 24.508 | 499.835 |
| CAP (dB/m) | 0.013 (0.008, 0.019) | 0.003 | <0.001 | 0.043 | 22.365 | 501.883 |
| Ballooning | | | | | | |
| FAST | 5.016 (3.786, 6.246) | 0.628 | <0.001 | 0.141 | 74.380 | 460.448 |
| LS (kPa) | 0.155 (0.099, 0.211) | 0.029 | <0.001 | 0.073 | 38.573 | 496.748 |
| NFS | 0.335 (0.196, 0.473) | 0.071 | <0.001 | 0.045 | 23.533 | 508.627 |
| FIB-4 | 0.499 (0.272, 0.726) | 0.116 | <0.001 | 0.042 | 22.393 | 512.896 |
| APRI | 1.248 (0.707, 1.788) | 0.276 | <0.001 | 0.050 | 26.349 | 509.022 |
| CAP (dB/m) | 0.004 (-0.002, 0.009) | 0.003 | 0.163 | 0.004 | 1.913 | 533.408 |
| Lobular inflammation | | | | | | |
| FAST | 4.661 (3.429, 5.894) | 0.629 | <0.001 | 0.116 | 62.683 | 485.048 |
| LS (kPa) | 0.095 (0.051, 0.140) | 0.023 | <0.001 | 0.035 | 19.026 | 528.820 |
| NFS | 0.175 (0.047, 0.304) | 0.066 | 0.007 | 0.012 | 6.576 | 539.855 |
| FIB-4 | 0.186 (0.007, 0.364) | 0.091 | 0.041 | 0.007 | 3.646 | 544.201 |
| APRI | 1.038 (0.610, 1.466) | 0.218 | <0.001 | 0.040 | 21.482 | 526.307 |
| CAP (dB/m) | 0.006 (0.000, 0.011) | 0.003 | 0.046 | 0.007 | 3.577 | 544.268 |
| Activity (Ballooning + Lobular inflammation) | | | | | | |
| FAST | 5.359 (4.190, 6.529) | 0.597 | <0.001 | 0.120 | 90.399 | 673.033 |
| LS (kPa) | 0.127 (0.080, 1.175) | 0.000 | <0.001 | 0.047 | 35.152 | 728.749 |
| NFS | 0.269 (0.143, 0.395) | 0.064 | <0.001 | 0.023 | 17.366 | 741.530 |
| FIB-4 | 0.350 (0.163, 0.538) | 0.096 | <0.001 | 0.019 | 14.493 | 749.394 |
| APRI | 1.210 (0.733, 1.686) | 0.243 | <0.001 | 0.040 | 30.047 | 733.858 |
| CAP (dB/m) | 0.005 (0.000, 0.010) | 0.003 | 0.690 | 0.004 | 3.053 | 760.848 |
| Fibrosis | | | | | | |
| FAST | 4.531 (3.326, 5.736) | 0.615 | <0.001 | 0.090 | 58.569 | 601.901 |
| LS (kPa) | 0.321 (0.256, 0.385) | 0.033 | <0.001 | 0.196 | 127.189 | 533.289 |
| NFS | 0.538 (0.392, 0.684) | 0.074 | <0.001 | 0.080 | 51.918 | 607.346 |
| FIB-4 | 0.719 (0.505, 0.933) | 0.109 | <0.001 | 0.069 | 44.702 | 615.779 |
| APRI | 0.799 (0.041, 1.187) | 0.198 | <0.001 | 0.024 | 15.826 | 644.716 |
| Abbreviations: CI, confidence interval; SE, standard error; *R^2^*, R-squared value; AIC, Akaike information criterion; FAST, FibroScan-aspartate aminotransferase (AST) score; CAP, controlled attenuation parameter; LS, liver stiffness; NFS, nonalcoholic fatty liver disease fibrosis score; FIB-4, fibrosis index based on four factors; APRI, AST to platelet ratio index | | | | | | |
